# 3,4-Dihy­droxy­benzoic acid pyridine monosolvate

**DOI:** 10.1107/S1600536810047082

**Published:** 2010-11-20

**Authors:** Li-Cai Zhu

**Affiliations:** a School of Chemistry and the Environment, South China Normal University, Guangzhou 510631, People’s Republic of China

## Abstract

The asymmetric unit of the title compound, C_7_H_6_O_4_·C_5_H_5_N, consists of one 3,4-dihy­droxy­benzoic acid and one pyridine mol­ecule, both located on general positions. The 3,4-dihy­droxy­benzoic acid mol­ecules are arranged in layers and are connected by inter­molecular O—H⋯O hydrogen bonding, forming channels along the *a* axis in which the pyridine mol­ecules are located. The pyridine and the acid mol­ecules are additionally linked by strong O—H⋯N hydrogen bonding and by weak π–π stacking inter­actions with centroid–centroid distances between the pyridine rings of 3.727 (2) Å.

## Related literature

For related structures see: Aitipamula & Nangia (2005 ▶); Mazurek *et al.* (2007 ▶).
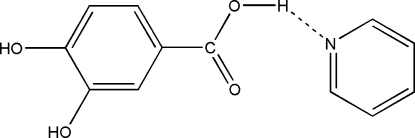


## Experimental

### 

#### Crystal data

C_7_H_6_O_4_·C_5_H_5_N
*M* *_r_* = 233.22
Monoclinic, 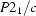

*a* = 11.9907 (11) Å
*b* = 9.1400 (8) Å
*c* = 10.3541 (9) Å
β = 108.042 (1)°
*V* = 1078.96 (17) Å^3^
*Z* = 4
Mo *K*α radiation
μ = 0.11 mm^−1^
*T* = 296 K
0.30 × 0.28 × 0.26 mm

#### Data collection

Bruker APEXII area-detector diffractometer
5415 measured reflections
1939 independent reflections
1484 reflections with *I* > 2σ(*I*)
*R*_int_ = 0.028

#### Refinement

*R*[*F* ^2^ > 2σ(*F* ^2^)] = 0.039
*wR*(*F* ^2^) = 0.107
*S* = 1.05
1939 reflections
157 parameters
H-atom parameters constrained
Δρ_max_ = 0.19 e Å^−3^
Δρ_min_ = −0.20 e Å^−3^

### 

Data collection: *APEX2* (Bruker, 2004 ▶); cell refinement: *SAINT* (Bruker, 2004 ▶); data reduction: *SAINT*; program(s) used to solve structure: *SHELXS97* (Sheldrick, 2008 ▶); program(s) used to refine structure: *SHELXL97* (Sheldrick, 2008 ▶); molecular graphics: *XP* in *SHELXTL* (Sheldrick, 2008 ▶); software used to prepare material for publication: *SHELXL97*.

## Supplementary Material

Crystal structure: contains datablocks I, global. DOI: 10.1107/S1600536810047082/nc2201sup1.cif

Structure factors: contains datablocks I. DOI: 10.1107/S1600536810047082/nc2201Isup2.hkl

Additional supplementary materials:  crystallographic information; 3D view; checkCIF report

## Figures and Tables

**Table 1:** Hydrogen-bond geometry (Å, °)

*D*—H⋯*A*	*D*—H	H⋯*A*	*D*⋯*A*	*D*—H⋯*A*
O1—H1*A*⋯O4^i^	0.82	1.95	2.6631 (17)	145
O2—H2⋯O1^ii^	0.82	1.95	2.7654 (16)	173
O3—H3⋯N1^iii^	0.82	1.77	2.5869 (19)	177

Symmetry codes: (i) 
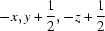
; (ii) 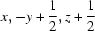; (iii) 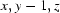.

**Fig. 1.**
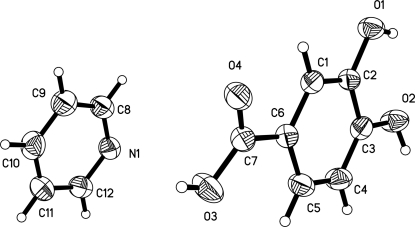
The molecular structure showing the atomic-numbering scheme and displacement ellipsoids drawn at the 50% probability level.

**Fig. 2.**
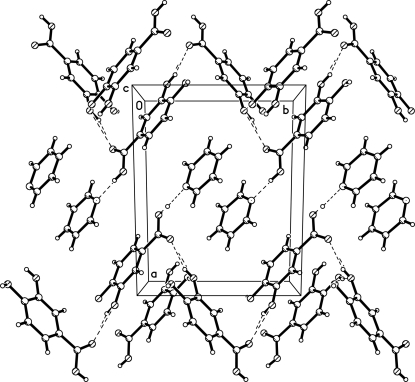
The molecular packing showing the intermolecular hydrogen bonding interactions as broken lines.

## supplementary crystallographic information

### Comment

The title compound was obtained unexpectedly in an unsuccessful attempt to prepare a 3,4-dihydroxybenzoate zinc complex. To identify the product a single crystal structure determination was performed. In the crystal structure of the title compound (Fig. 1), the 3,4-dihydroxybenzoic acid molecules are connected via intermolecular O—H···O hydrogen bonding into layers, that are located in the b-c-plane (Table 1 and Fig. 2). These layers are stacked in order that channels are formed, that elongate in the direction of the *b* axis. The channels are occupied by solvate molecules in a manner similar to that observed previously in related structure (Aitipamula & Nangia, 2005; Mazurek *et al.*, 2007). The pyridine solvate molecules within the channels are connected to the acid molecules by O—H···N hydrogen bonding and by weak π-π stacking interactions with centroid-to-centroid distances between related pyridine rings of 3.727 (2)Å (Fig. 2).

### Experimental

The compound was obtained unexpectedly in an unsuccessful attempt to prepare a 3,4-dihydroxybenzoate zinc complex. A mixture of 3,4-dihydroxybenzoic acid (0.31 g, 2 mmol), zinc chloride (0.136 g, 1 mmol) and pyridine (0.16 ml, 2 mmol) was stirred with methanol (15 ml) for 0.5 h at room temperature. Several days later, colorless block crystals suitable for X-ray analysis were obtained by slow evaporation of the mixed solution.

### Refinement

All H atoms were placed at calculated positions (O-H H atoms allowed to rotate but not to tip and were treated as riding,  with C—H = 0.93 and O—H = 0.82 Å, and with *U*_iso_(H) = 1.2 or 1.5 *U*_eq_(C, O).

### Crystal data

**Table:**

C_7_H_6_O_4_·C_5_H_5_N	*F*(000) = 488
*M**_r_* = 233.22	*D*_x_ = 1.436 Mg m^−^^3^
Monoclinic, *P*2_1_/*c*	Mo *K*α radiation, λ = 0.71073 Å
Hall symbol: -P 2ybc	Cell parameters from 1420 reflections
*a* = 11.9907 (11) Å	θ = 2.9–25.4°
*b* = 9.1400 (8) Å	µ = 0.11 mm^−^^1^
*c* = 10.3541 (9) Å	*T* = 296 K
β = 108.042 (1)°	Block, colorless
*V* = 1078.96 (17) Å^3^	0.30 × 0.28 × 0.26 mm
*Z* = 4	

### Data collection

**Table:**

Bruker APEXII area-detector diffractometer	1484 reflections with *I* > 2σ(*I*)
Radiation source: fine-focus sealed tube	*R*_int_ = 0.028
graphite	θ_max_ = 25.2°, θ_min_ = 1.8°
f and ω scan	*h* = −6→14
5415 measured reflections	*k* = −10→10
1939 independent reflections	*l* = −12→12

### Refinement

**Table:**

Refinement on *F*^2^	Primary atom site location: structure-invariant direct methods
Least-squares matrix: full	Secondary atom site location: difference Fourier map
*R*[*F*^2^ > 2σ(*F*^2^)] = 0.039	Hydrogen site location: inferred from neighbouring sites
*wR*(*F*^2^) = 0.107	H-atom parameters constrained
*S* = 1.05	*w* = 1/[σ^2^(*F*_o_^2^) + (0.0507*P*)^2^ + 0.1871*P*] where *P* = (*F*_o_^2^ + 2*F*_c_^2^)/3
1939 reflections	(Δ/σ)_max_ < 0.001
157 parameters	Δρ_max_ = 0.19 e Å^−^^3^
0 restraints	Δρ_min_ = −0.20 e Å^−^^3^

### Special details

**Table:**

Geometry. All e.s.d.'s (except the e.s.d. in the dihedral angle between two l.s. planes) are estimated using the full covariance matrix. The cell e.s.d.'s are taken into account individually in the estimation of e.s.d.'s in distances, angles and torsion angles; correlations between e.s.d.'s in cell parameters are only used when they are defined by crystal symmetry. An approximate (isotropic) treatment of cell e.s.d.'s is used for estimating e.s.d.'s involving l.s. planes.
Refinement. Refinement of *F*^2^ against ALL reflections. The weighted *R*-factor *wR* and goodness of fit *S* are based on *F*^2^, conventional *R*-factors *R* are based on *F*, with *F* set to zero for negative *F*^2^. The threshold expression of *F*^2^ > σ(*F*^2^) is used only for calculating *R*-factors(gt) *etc*. and is not relevant to the choice of reflections for refinement. *R*-factors based on *F*^2^ are statistically about twice as large as those based on *F*, and *R*- factors based on ALL data will be even larger.

### Fractional atomic coordinates and isotropic or equivalent isotropic displacement parameters (Å^2^)

**Table:**

	*x*	*y*	*z*	*U*_iso_*/*U*_eq_	
C1	0.10603 (14)	0.03303 (18)	0.32574 (17)	0.0312 (4)	
H1	0.0994	−0.0100	0.2423	0.037*	
C2	0.02226 (14)	0.13119 (18)	0.33621 (16)	0.0298 (4)	
C3	0.03141 (15)	0.19523 (18)	0.46141 (17)	0.0325 (4)	
C4	0.12446 (16)	0.1597 (2)	0.57352 (17)	0.0386 (4)	
H4	0.1305	0.2020	0.6571	0.046*	
C5	0.20928 (15)	0.0613 (2)	0.56300 (17)	0.0377 (4)	
H5	0.2719	0.0382	0.6392	0.045*	
C6	0.20063 (14)	−0.00256 (18)	0.43869 (16)	0.0307 (4)	
C7	0.28879 (14)	−0.11003 (19)	0.42301 (17)	0.0329 (4)	
C8	0.50997 (17)	0.6408 (2)	0.35399 (19)	0.0449 (5)	
H8	0.4397	0.6633	0.2877	0.054*	
C9	0.58295 (18)	0.5384 (2)	0.3254 (2)	0.0503 (5)	
H9	0.5626	0.4930	0.2408	0.060*	
C10	0.68609 (18)	0.5041 (2)	0.4233 (2)	0.0500 (5)	
H10	0.7361	0.4339	0.4067	0.060*	
C11	0.71475 (17)	0.5750 (2)	0.5466 (2)	0.0496 (5)	
H11	0.7847	0.5545	0.6142	0.060*	
C12	0.63792 (17)	0.6765 (2)	0.56739 (19)	0.0455 (5)	
H12	0.6573	0.7247	0.6505	0.055*	
O1	−0.06869 (10)	0.16177 (13)	0.22200 (11)	0.0382 (3)	
H1A	−0.1153	0.2163	0.2415	0.057*	
O2	−0.05466 (11)	0.29250 (14)	0.46347 (12)	0.0425 (3)	
H2	−0.0526	0.3074	0.5423	0.064*	
O3	0.38824 (10)	−0.10876 (15)	0.51991 (13)	0.0486 (4)	
H3	0.4329	−0.1683	0.5030	0.073*	
O4	0.26885 (10)	−0.19100 (13)	0.32362 (12)	0.0384 (3)	
N1	0.53651 (13)	0.70907 (16)	0.47356 (15)	0.0406 (4)	

### Atomic displacement parameters (Å^2^)

**Table:**

	*U*^11^	*U*^22^	*U*^33^	*U*^12^	*U*^13^	*U*^23^
C1	0.0317 (9)	0.0346 (9)	0.0286 (9)	−0.0025 (8)	0.0110 (7)	−0.0007 (7)
C2	0.0274 (9)	0.0338 (9)	0.0265 (8)	−0.0004 (7)	0.0060 (7)	0.0050 (7)
C3	0.0339 (9)	0.0334 (9)	0.0329 (9)	0.0022 (8)	0.0140 (8)	0.0023 (7)
C4	0.0411 (10)	0.0464 (10)	0.0272 (9)	0.0039 (9)	0.0089 (8)	−0.0040 (8)
C5	0.0326 (10)	0.0453 (10)	0.0309 (9)	0.0041 (8)	0.0039 (8)	0.0002 (8)
C6	0.0278 (9)	0.0340 (9)	0.0299 (9)	−0.0015 (7)	0.0086 (7)	0.0016 (7)
C7	0.0287 (9)	0.0366 (9)	0.0323 (9)	−0.0012 (8)	0.0081 (8)	0.0032 (7)
C8	0.0391 (11)	0.0522 (12)	0.0403 (11)	0.0056 (9)	0.0075 (9)	−0.0002 (9)
C9	0.0507 (12)	0.0546 (12)	0.0460 (11)	0.0056 (10)	0.0158 (10)	−0.0064 (10)
C10	0.0480 (12)	0.0494 (12)	0.0568 (13)	0.0128 (10)	0.0225 (11)	0.0041 (10)
C11	0.0397 (11)	0.0587 (13)	0.0477 (12)	0.0116 (10)	0.0093 (9)	0.0084 (10)
C12	0.0424 (11)	0.0538 (12)	0.0376 (10)	0.0046 (10)	0.0087 (9)	−0.0006 (9)
O1	0.0351 (7)	0.0481 (8)	0.0290 (6)	0.0102 (6)	0.0065 (6)	0.0007 (5)
O2	0.0464 (8)	0.0500 (8)	0.0311 (7)	0.0165 (6)	0.0122 (6)	0.0008 (6)
O3	0.0322 (7)	0.0618 (9)	0.0437 (8)	0.0133 (7)	0.0001 (6)	−0.0137 (6)
O4	0.0309 (7)	0.0457 (7)	0.0363 (7)	0.0017 (6)	0.0072 (5)	−0.0079 (6)
N1	0.0361 (9)	0.0441 (9)	0.0409 (9)	0.0054 (7)	0.0109 (7)	0.0005 (7)

### Geometric parameters (Å, °)

**Table:**

C1—C2	1.376 (2)	C8—N1	1.334 (2)
C1—C6	1.392 (2)	C8—C9	1.375 (3)
C1—H1	0.9300	C8—H8	0.9300
C2—O1	1.3664 (18)	C9—C10	1.371 (3)
C2—C3	1.395 (2)	C9—H9	0.9300
C3—O2	1.367 (2)	C10—C11	1.377 (3)
C3—C4	1.377 (2)	C10—H10	0.9300
C4—C5	1.387 (2)	C11—C12	1.371 (3)
C4—H4	0.9300	C11—H11	0.9300
C5—C6	1.388 (2)	C12—N1	1.334 (2)
C5—H5	0.9300	C12—H12	0.9300
C6—C7	1.488 (2)	O1—H1A	0.8200
C7—O4	1.2294 (19)	O2—H2	0.8200
C7—O3	1.299 (2)	O3—H3	0.8200
			
C2—C1—C6	120.72 (15)	O3—C7—C6	115.02 (15)
C2—C1—H1	119.6	N1—C8—C9	122.25 (18)
C6—C1—H1	119.6	N1—C8—H8	118.9
O1—C2—C1	118.14 (14)	C9—C8—H8	118.9
O1—C2—C3	122.02 (15)	C10—C9—C8	118.97 (19)
C1—C2—C3	119.83 (15)	C10—C9—H9	120.5
O2—C3—C4	123.97 (15)	C8—C9—H9	120.5
O2—C3—C2	116.41 (15)	C9—C10—C11	119.18 (19)
C4—C3—C2	119.62 (15)	C9—C10—H10	120.4
C3—C4—C5	120.62 (16)	C11—C10—H10	120.4
C3—C4—H4	119.7	C12—C11—C10	118.50 (19)
C5—C4—H4	119.7	C12—C11—H11	120.7
C4—C5—C6	119.97 (16)	C10—C11—H11	120.7
C4—C5—H5	120.0	N1—C12—C11	122.82 (18)
C6—C5—H5	120.0	N1—C12—H12	118.6
C5—C6—C1	119.23 (15)	C11—C12—H12	118.6
C5—C6—C7	121.82 (15)	C2—O1—H1A	109.5
C1—C6—C7	118.94 (15)	C3—O2—H2	109.5
O4—C7—O3	123.08 (16)	C7—O3—H3	109.5
O4—C7—C6	121.87 (15)	C8—N1—C12	118.25 (16)

### Hydrogen-bond geometry (Å, °)

**Table:**

*D*—H···*A*	*D*—H	H···*A*	*D*···*A*	*D*—H···*A*
O1—H1A···O4^i^	0.82	1.95	2.6631 (17)	145
O2—H2···O1^ii^	0.82	1.95	2.7654 (16)	173
O3—H3···N1^iii^	0.82	1.77	2.5869 (19)	177

Symmetry codes: (i) −*x*, *y*+1/2, −*z*+1/2; (ii) *x*, −*y*+1/2, *z*+1/2; (iii) *x*, *y*−1, *z*.
